# Portable ultra‐low‐field magnetic resonance imaging enables postictal seizure imaging

**DOI:** 10.1111/epi.18273

**Published:** 2025-02-17

**Authors:** Tobias Bauer, Hemmen Sabir, Tobias Baumgartner, Attila Rácz, Jan Pukropski, Mostafa Badr, Simon Olbrich, Annalena Lange, Justus Bisten, Anne Groteklaes, Nils C. Lehnen, Fernando Cendes, Alexander Radbruch, Rainer Surges, Theodor Rüber

**Affiliations:** ^1^ Department of Neuroradiology University Hospital Bonn Bonn Germany; ^2^ Department of Epileptology University Hospital Bonn Bonn Germany; ^3^ German Center for Neurodegenerative Diseases (DZNE) Bonn Germany; ^4^ Department of Neonatology and Pediatric Intensive Care University Hospital Bonn Bonn Germany; ^5^ Institute for Computer Science University of Bonn Bonn Germany; ^6^ Department of Neurology University of Campinas Campinas Brazil; ^7^ Center for Medical Data Usability and Translation University of Bonn Bonn Germany

**Keywords:** cortical localization, diffusion imaging, epilepsy surgery, functional neuroimaging, global epileptology

## Abstract

The detection of transient peri‐ictal magnetic resonance imaging (MRI) abnormalities has been variable after epileptic seizures. The most common reason for this variability is that abnormalities may disappear if the interval between seizure and scan acquisition is prolonged using conventional high‐field systems. Here, we deployed a portable ultra‐low‐field MRI system in the presurgical evaluation at the bedside of individuals with epilepsy. We hypothesized that this novel technology enables rapid postictal scans and reliably shows focal peri‐ictal MRI abnormalities in the seizure onset zone. A .064‐T Swoop Portable MR Imaging System was used. Postictally, an axial diffusion‐weighted sequence was acquired. The interictal MRI consisted of the diffusion‐weighted and three‐dimensional T1‐weighted sequences. Postictal–interictal difference maps of diffusion‐weighted volumes were calculated. Three individuals were included. Two individuals with focal aware seizures scanned 29 s and 19 min after the seizure, respectively, showed focal restrictions in diffusivity in the seizure onset zone, and a third individual scanned 5 h 45 min after a focal to bilateral tonic–clonic seizure showed global restrictions of diffusivity. Portable ultra‐low‐field MRI opens a new line of inquiry with the aim to establish postictal seizure imaging as part of the presurgical evaluation of people with epilepsy.

## 1 INTRODUCTION

Epilepsy imaging is geared toward identifying a potentially epileptogenic lesion; the visualization of epileptic seizures, despite being the pathognomonic symptom of epilepsy, plays little role.^1^ As epileptic seizures may not directly be observed in a magnetic resonance imaging (MRI) scanner for pragmatic reasons, the idea is to use MRI to post hoc visualize microstructural processes anatomically and temporally related to individual seizures.^2^ The two processes on which MRI research has focused most so far are peri‐ictal blood–brain barrier dysfunction^3^ and peri‐ictal alterations in perfusion.^4^ Among the MRI biomarkers representing these processes are diffusivity,^5^ enhancement of gadolinium‐based contrast agent,^6^ susceptibility,^7^ and regional cerebral blood flow.^8^ To date, ictal single photon emission computed tomography (SPECT) is the only clinically established imaging modality for the visualization of single epileptic seizures. The procedure is economically costly and time intensive and exposes individuals to an average radiation dose of 6.8 mSv.^9^ Furthermore, it has only a moderate diagnostic sensitivity of approximately 50%.^10^ Extensive research has aimed to establish postictal diffusivity changes as markers of prior ictal activity, with alterations in the apparent diffusion coefficient (ADC) being most commonly reported. However, colocalization with the presumed seizure onset zone has been variable, occurring in only one third of cases.^11^ The most common reason given for this variability is that imaging sequelae may disappear if the interval between seizure and scan acquisition is prolonged. Recent advancements in portable ultra‐low‐field (ULF) MRI technology offer the chance for nearly instant imaging at the bedside.^12^ Here, we argue that the advent of portable ULF‐MRI heralds new opportunities for postictal seizure imaging. It is our rationale that postictal diffusion may not have been reliably measured, because the seizure–MRI latency is too long when conventional high‐field systems are used. Presenting three cases, we aim to showcase that postictal restrictions in diffusivity can be shown focally using portable ULF‐MRI.

## 2 MATERIALS AND METHODS

### 2.1 Study design

Postictal and interictal ULF‐MRI was performed at the Department of Epileptology, University Hospital Bonn. We aimed to perform a postictal ULF‐MRI with a minimal latency between a seizure and the start of the ULF‐MRI scan. Adult individuals with epilepsy who were admitted to our video‐electroencephalographic (video‐EEG) unit as part of their presurgical evaluation were screened for eligibility (no contraindications for ULF‐MRI, such as pacemakers). When they had an EEG‐confirmed seizure, the portable MRI was brought to their room, and EEG electrodes were removed immediately after termination of the seizure. The individual's head was placed in the MRI while they remained in their bed. The study was approved by the institutional review board of the University Hospital Bonn. All participants provided written informed consent.

### 2.2 ULF‐MRI protocol

ULF‐MRI scans were acquired using a .064‐T Swoop Portable MR Imaging System (Hyperfine). The serial measurement of at least two diffusion‐weighted images with different diffusion weighting is needed to determine the ADC quantitatively. Postictally, an axial diffusion‐weighted sequence (b = 0 s/mm^2^ and b = 900 s/mm^2^) was acquired (in‐plane resolution = 2.4 mm, matrix = 76 × 92, slice thickness = 5.9 mm, 34 slices, scanning time = 10 min 21 s). The interictal MRI consisted of an axial diffusion‐weighted sequence, axial fluid‐attenuated inversion recovery, three‐dimensional T1‐weighted, and T2‐weighted sequences (total scanning time = 39 min 10 s). The protocol was provided by the manufacturer, and sequences correspond to the sequences described in a previous publication.^13^ Sequence details can be found in Table 1.

**TABLE 1:** Scanning protocol used for the ultra‐low‐field magnetic resonance imaging.

Sequence	Orientation	In‐plane resolution, mm	Slice thickness, mm	TR, ms	TE, ms	TI, ms	Duration, min:s
T1w^a^	3D	2.2	2.2	1000	4.75	270	9:46
T2w^a^	3D	2.2	2.2	2000	148.16	na	10:04
FLAIR^a^	Axial	1.7	5	3500	162.18	1301.11	8:59
DWI	Axial	2.4	5.9	1000	80.3	na	10:21

Abbreviations: 3D, three‐dimensional; DWI, diffusion‐weighted imaging; FLAIR, fluid‐attenuated inversion recovery; na, not applicable; T1w, T1‐weighted; T2w, T2‐weighted; TE, echo time; TI, inversion time; TR, repetition time.

^a^ Interictal scan only.

### 2.3 Image analysis

Postictal diffusion‐weighted volumes were coregistered to the respective interictal diffusion‐weighted scans.^14^ In interictal space, postictal and interictal images were smoothed with a Gaussian kernel of 5‐mm full width at half maximum to increase the signal‐to‐noise ratio before the postictal–interictal difference maps were calculated. For an anatomical representation, a 1‐mm isotropic T1‐weighted image was synthesized from the interictal T1‐ and T2‐weighted scans using a superresolution approach (Figure S1).^15^ A threshold of ΔADC < −200 ⋅ 10^−6^ mm^2^/s was applied to the postictal–interictal difference maps. For visualization, difference maps were resampled to the 1‐mm isotropic voxel space of the synthesized T1‐weighted image using linear interpolation.

## 3 RESULTS

Three individuals with epilepsy were scanned postictally and interictally. Seizure types included two focal aware seizures (Cases 1 and 2) and one focal to bilateral tonic–clonic seizure (Case 3). The seizure–MRI latency varied from 29 s (Case 1) to 5 h 45 min (Case 3). There were no complications or adverse events during the deployment of the ULF‐MRI.

### 3.1 Case 1

**FIGURE 1:**
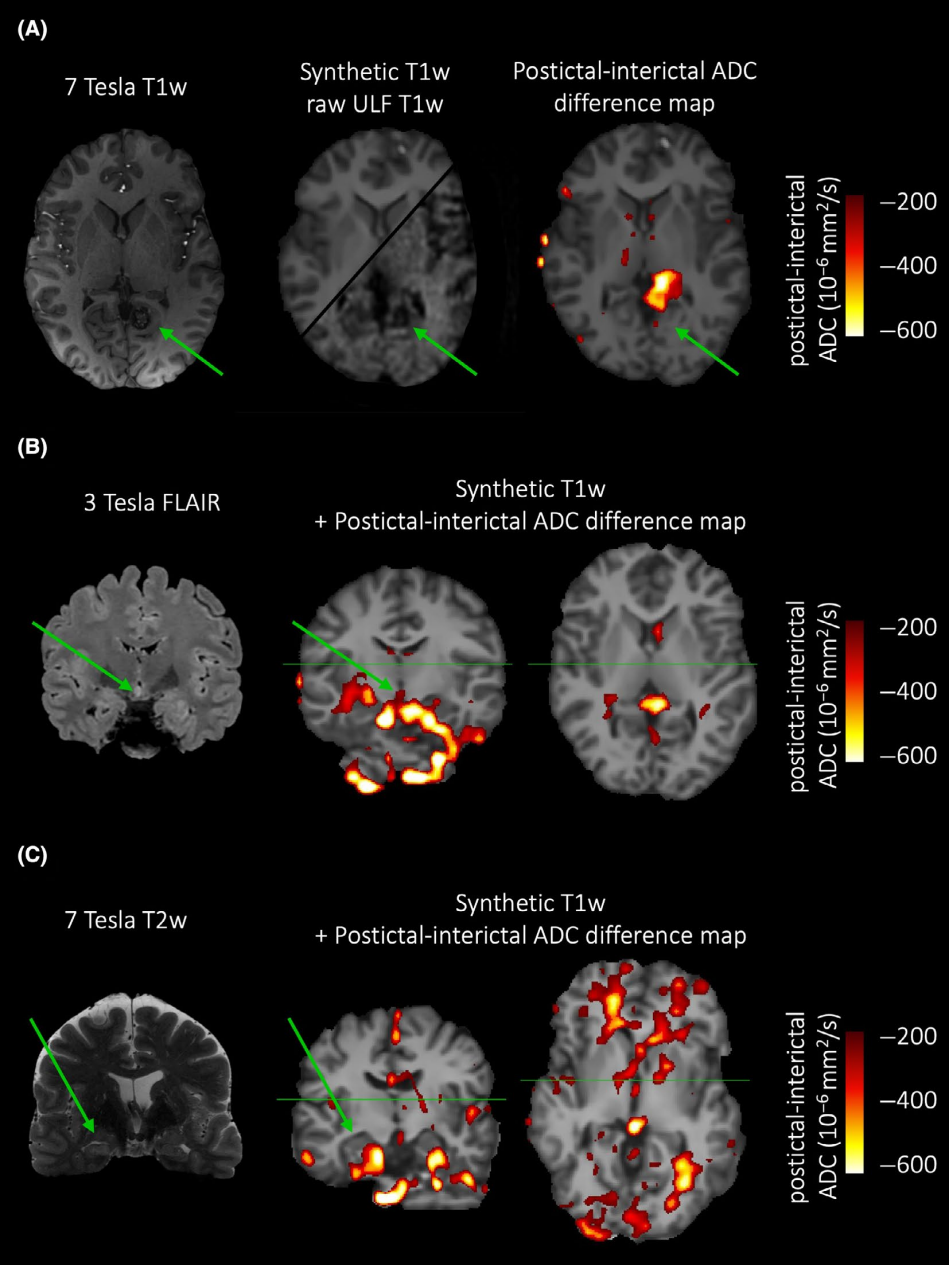
(A) Case 1: 31‐year‐old female with left occipital cavernoma with focal aware seizures; seizure‐scan latency: 29 s. (B) Case 2: 18‐year‐old female with tuber cinereum hamartoma and focal aware gelastic seizures; seizure‐scan latency: 19 min. Please note the postictal diffusion restriction in the splenium of the corpus callosum (right image). (C) Case 3: 53‐year‐old male with right hippocampal sclerosis after a focal to bilateral tonic–clonic seizure; seizure‐scan latency: 5 h 43 min. See Figure S2 for postictal–interictal difference maps with higher and lower ΔADC‐thresholds. ADC, apparent diffusion coefficient; FLAIR, fluid‐attenuated inversion recovery; T1w, T1‐weighted; T2w, T2‐weighted; ULF, ultra‐low‐field.

A 31‐year‐old female with left occipital cavernoma and focal aware seizures presented with oral automatisms (swallowing, lip smacking, eye‐blinking). EEG showed a beginning with occipital lambda waves, evolution of rhythmic theta activity, and then a generalized seizure pattern. The duration of this seizure was 1 min 41 s. She had five more stereotypic seizures within the preceding 24 h while in the video‐EEG monitoring unit. The postictal MRI scan was started 29 s after the termination of this last seizure, and she had another stereotypic seizure during adjustment measurements immediately preceding the diffusion‐weighted sequence. The interictal MRI was performed after a seizure‐free interval of 22 h 3 min. Postictal–interictal ADC maps showed a postictal diffusion restriction in the anatomical vicinity of the cavernoma (green arrows) as a likely epileptogenic lesion (Figure 1A).

### 3.2 Case 2

An 18‐year‐old female presented with tuber cinereum hamartoma and focal aware gelastic seizures. The EEG showed no changes, thus the seizure ending point was clinically determined as the cessation of laughter. The seizure duration was approximately 15 s, and two more gelastic seizures were recorded within the preceding 36 h at our video‐EEG monitoring unit. The postictal scan was started 19 min after the last seizure. The interictal MRI had been performed 36 h before the postictal scan, after a reported seizure‐free interval of 6 days. Postictal–interictal ADC maps showed a postictal diffusion restriction in the anatomical vicinity of the tuber cinereum hamartoma as a likely epileptogenic lesion (Figure 1B). In addition, postictal diffusion restriction was seen in the splenium of the corpus callosum (Figure 1B, right).

### 3.3 Case 3

A 53‐year‐old male presented with right hippocampal sclerosis. He had both focal aware and focal to bilateral tonic–clonic seizures. The earlier seizure was a focal to bilateral tonic–clonic seizure of approximately 5‐min duration. EEG showed an interictal right temporal focus, bilateral rhythmic theta activity, and a seizure pattern that rapidly generalized. The postictal scan was performed 5 h 43 min after termination of the seizure. The interictal MRI was performed 6 days later, after a seizure‐free interval of >36 h. ADC maps show a generalized postictal diffusion restriction (Figure 1C). However, a lower threshold resulted in a remaining prominent cluster in the right hippocampus (Figure S2C, right).

## 4 DISCUSSION

With this case series, we demonstrate the feasibility of postictal portable ULF‐MRI as part of the presurgical evaluation in individuals with epilepsy. Postictal lower ADC decrease was shown 29 s to >5 h after termination of the seizure at the bedside. The advent of portable ULF‐MRI opens a new window for postictal seizure imaging.

The general occurrence of restrictions in diffusivity as indicated by lower ADC after seizures has long been known. It is assumed that postictal dynamic fluctuations of diffusivity show initial hyperperfusion (higher ADC) followed by vasogenic extracellular edema (lower ADC) and finally followed by cytotoxic edema (higher ADC).^11^ A diffusion restriction in the splenium of the corpus callosum immediately following a seizure, as seen in Case 2, has also been previously reported.^16^ However, until now, it has only been speculative whether localized alterations in diffusivity reliably occur in the anatomical vicinity of the presumed seizure when MRI is conducted minutes after the seizures. Due to advancements in technology, this pilot study provides initial evidence for this. Case 1, who underwent ULF‐MRI seconds after seizure termination, illustrates a clear focal finding localized around the likely epileptogenic lesion (occipital cavernoma). In Case 2, minutes after the seizure ended, additional findings were observed not only near the likely epileptogenic lesion (hypothalamic hamartoma) but also in the cerebellum and various cortical regions. Given our limited data and experience with this new protocol, we cannot yet determine whether these findings represent artifacts (false positive) or are a physiological result of the extended interval between seizure and scan (true positive). We regard the pattern observed at a single threshold in Case 3 as nonlocalizable and speculate that this may be due to both the prolonged seizure–scan latency and the bilateral seizure pattern/generalization of the seizure. However, the observation that the cluster near the epileptogenic lesion (right hippocampal sclerosis) remained most prominent at a lower threshold suggests that varying thresholds could aid in enhancing localization.

The potential advantages of postictal portable ULF‐MRI compared to ictal SPECT are obvious, including the lack of radiation exposure, convenient bedside application, and reduced costs. However, future studies need to validate its potential clinical significance in a prospective study with a higher sample size. Specifically, future studies should address the following key questions. First, the optimal time window for detecting postictal diffusion changes needs to be defined. To achieve this, longitudinal imaging is essential to establish the time course of postictal diffusion changes. Second, it should be clarified whether diffusivity restrictions also allow focal localization after focal to bilateral tonic–clonic seizures or whether diffusivity restrictions are global. Third, an optimal localization paradigm rather than simple thresholding at a single value should be developed. Finally, the diagnostic yield and localization accuracy should be quantified in comparison to established diagnostic modalities, such as high‐field MRI, SPECT, positron emission tomography, and EEG.

All in all, portable ULF‐MRI, originally developed to bridge the gap in diagnostic capabilities between well‐resourced and limited‐resource settings, represents a major opportunity in the decades‐long quest to realize postictal seizure imaging on clinical grounds.

## CONFLICT OF INTEREST STATEMENT

A.Rá. has received speaker fees from UCB Pharma and has received travel support from the Elisabeth und Helmut Uhl Stiftung. A.Ra. serves on the scientific advisory boards for GE Healthcare, Bracco, Bayer, Guerbet, and AbbVie; has received speaker honoraria from Bayer, Guerbet, Siemens, and Medscape; and is a consultant for, and has received institutional study support from, Guerbet and Bayer. R.S. has received personal fees as a speaker or for serving on advisory boards from Angelini, Arvelle, Bial, Desitin, Eisai, Jazz Pharmaceuticals Germany, Janssen‐Cilag, LivaNova, LivAssured, Novartis, Precisis, Rapport Therapeutics, Tabuk Pharmaceuticals, UCB Pharma, UNEEG, and Zogenix. He is an editorial board member of *Epilepsy and Behavior* and associate editor of *Epilepsia Open*. These activities were not related to the content of this article. None of the other authors has any conflict of interest to disclose. We confirm that we have read the Journal's position on issues involved in ethical publication and affirm that this report is consistent with those guidelines.

## Supporting information


FIGURES S1–S2.

## Data Availability

The data that support the findings of this study are available on request from the corresponding author. The data are not publicly available due to their containing information that could compromise the privacy of research participants.
