# Supplementary material for: Portable ultra‐low‐field magnetic resonance imaging enables postictal seizure imaging
Source: Epilepsia. 2025 Feb 17;66(4):e60–5. doi: 10.1111/epi.18273 (PMC11997907; doi:10.1111/epi.18273)
Supplement: Supplementary file 1 — FIGURES S1–S2. [file EPI-66-e60-s001.pdf]

Portable ultra-low-field MRI enables postictal seizure imaging

Authors: Tobias Bauer[1,2,3,*], Hemmen Sabir[3,4,*], Tobias Baumgartner[2], Attila Rácz[2], Jan Pukropski[2], Mostafa Badr[2], Simon Olbrich[1,2], Annalena Lange[1,2,5], Justus Bisten[1,2,5], Anne Groteklaes[4], Nils Lehnen[1], Fernando Cendes[6], Alexander Radbruch[1,3,7], Rainer Surges[2], Theodor Rüber[1,2,3,7]

Author affiliations: [1]Department of Neuroradiology, University Hospital Bonn, Bonn, Germany; [2]Department of Epileptology, University Hospital Bonn, Bonn, Germany; [3]German Center for Neurodegenerative Diseases (DZNE), Bonn, Germany; [4]Department of Neonatology and Pediatric Intensive Care, University Hospital Bonn, Bonn, Germany; [5]Institute for Computer Science, University of Bonn, Bonn, Germany; [6]Department of Neurology, University of Campinas, Campinas, Brazil; [7]Center for Medical Data Usability and Translation, University of Bonn, Bonn, Germany

[*]shared first authors

Corresponding author: Dr. Theodor Rüber, MD
Venusberg-Campus 1, 53127 Bonn, Germany
theodor.rueber@ukbonn.de; +49 228 6885-264

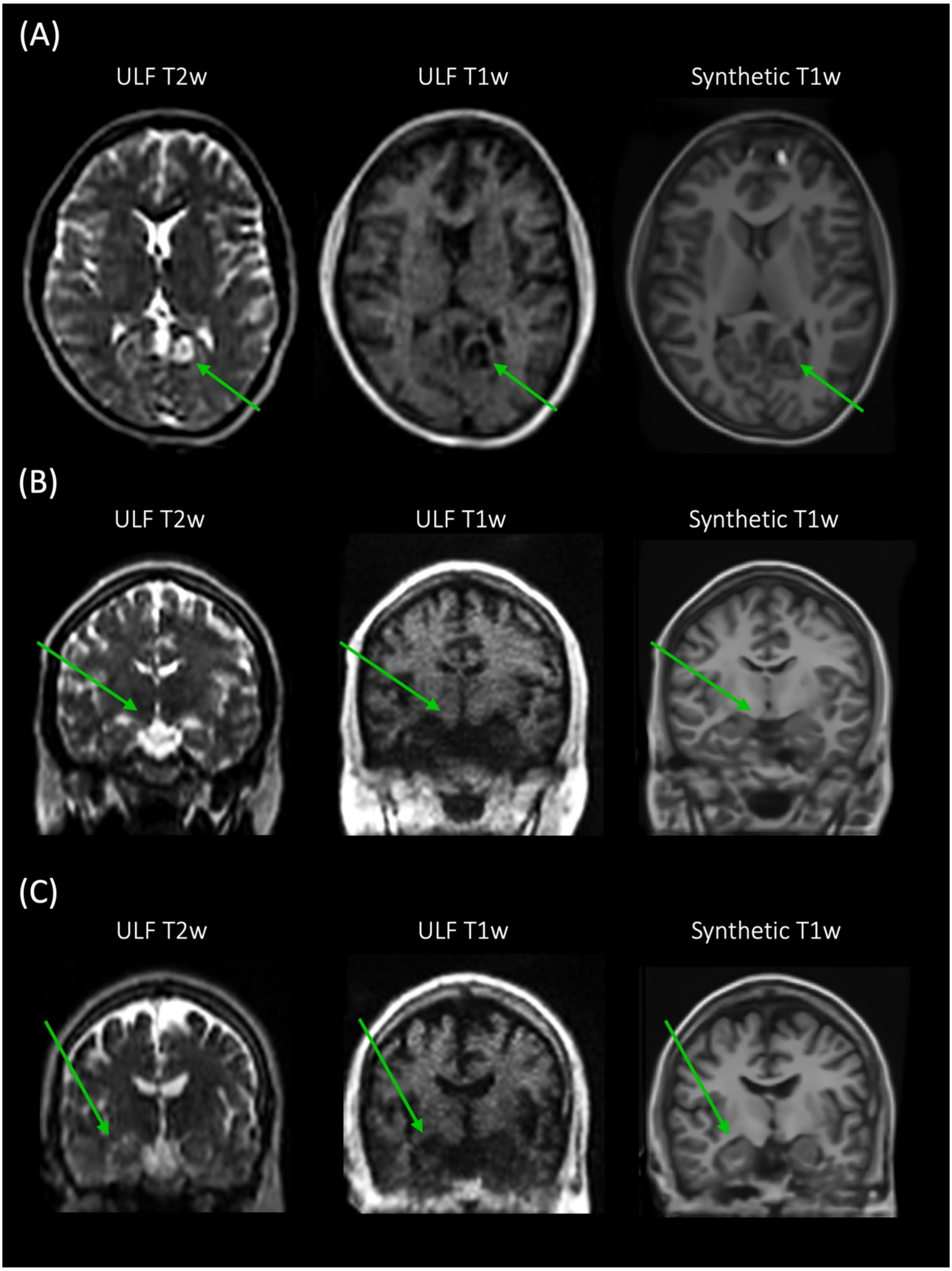

Supplementary Figure S1. Structural MRI scans for (A) case #1, (B) case #2 and (C) case #3. T2 weighted (T2w, left), T1 weighted (T1w, middle), and a synthetic 1 mm isotropic T1w scan generated using the SynthSR super-resolution approach included in FreeSurfer (Iglesias JE, Schleicher R, Laguna S, Billot B, Schaefer P, McKaig B, et al. Quantitative Brain Morphometry of Portable Low-Field-Strength MRI Using Super-Resolution Machine Learning. Radiology. 2023; 306(3)). ULF: ultra-low-field.

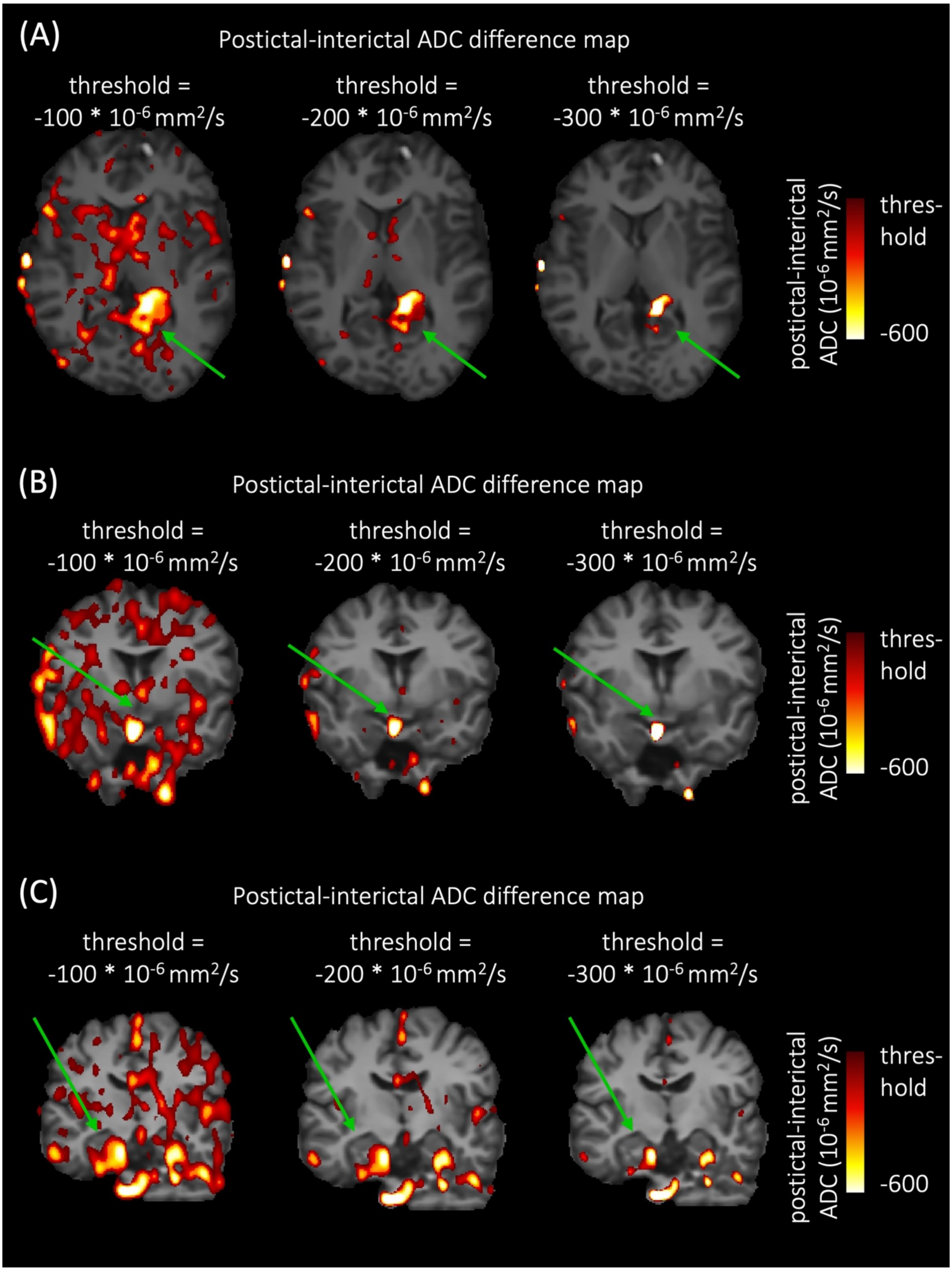

Supplementary Figure S2. Postictal-interictal ADC difference maps at different thresholds for (A) case #1, (B) case #2 and (C) case #3. ADC: apparent diffusion coefficient.
